# LGD: a comprehensive online database of Liliales based on multi-omics data

**DOI:** 10.1093/hr/uhaf311

**Published:** 2025-11-13

**Authors:** Sujuan Xu, Tian Zhang, Hanhan Feng, Ze Wu, Aiping Song, Nianjun Teng

**Affiliations:** Key Laboratory of Landscaping Agriculture/Key Laboratory of Flower Biology and Germplasm Innovation, Ministry of Agriculture and Rural Affairs, College of Horticulture, Nanjing Agricultural University, Nanjing 211800, China; Lily Science and Technology Backyard Qixia of Jiangsu, Nanjing 210043, China; Key Laboratory of Landscaping Agriculture/Key Laboratory of Flower Biology and Germplasm Innovation, Ministry of Agriculture and Rural Affairs, College of Horticulture, Nanjing Agricultural University, Nanjing 211800, China; Lily Science and Technology Backyard Qixia of Jiangsu, Nanjing 210043, China; Key Laboratory of Landscaping Agriculture/Key Laboratory of Flower Biology and Germplasm Innovation, Ministry of Agriculture and Rural Affairs, College of Horticulture, Nanjing Agricultural University, Nanjing 211800, China; Lily Science and Technology Backyard Qixia of Jiangsu, Nanjing 210043, China; Key Laboratory of Landscaping Agriculture/Key Laboratory of Flower Biology and Germplasm Innovation, Ministry of Agriculture and Rural Affairs, College of Horticulture, Nanjing Agricultural University, Nanjing 211800, China; Lily Science and Technology Backyard Qixia of Jiangsu, Nanjing 210043, China; Key Laboratory of Landscaping Agriculture/Key Laboratory of Flower Biology and Germplasm Innovation, Ministry of Agriculture and Rural Affairs, College of Horticulture, Nanjing Agricultural University, Nanjing 211800, China; Key Laboratory of Landscaping Agriculture/Key Laboratory of Flower Biology and Germplasm Innovation, Ministry of Agriculture and Rural Affairs, College of Horticulture, Nanjing Agricultural University, Nanjing 211800, China; Lily Science and Technology Backyard Qixia of Jiangsu, Nanjing 210043, China

Dear Editor,

The order Liliales, with approximately 10 families, 64 genera, and 1500 species, is mainly associated with bulbous or terrestrial habits; most members of this order have rhizomes, bulbs, or corms, holding significant economic importance as food sources, pharmaceutical resources, and ornamental plants. Liliales evolved giant genomes relative to other angiosperms but with significant variation, ranging from 1.5 to 147 Gb. With advances in high-throughput technologies, multi-omics data for Liliales species continue to accumulate [1–6]. However, the extremely large genomes and exceptionally long gene introns pose significant challenges for genomic analysis and utilization.

Several platforms have been developed for the utilization of multi-omics data for various plants [7–9]; however, there is currently no comprehensive platform for Liliales that combines multi-omics data and bioinformatics tools. Therefore, we developed the Liliales Genome Database (LGD; https://lgd.njau.edu.cn/) to enable efficient utilization of available genome data and advance Liliales fundamental research and genetic breeding. The current release of the LGD platform integrates genomic data from eight species within the order Liliales, totaling 117.43 Gb: *Lilium davidii* var. *unicolor*, *L. sargentiae*, *L. regale*, and *Calochortus tolmiei* (Liliaceae); *Gloriosa superba* (Colchicaceae); and *Chionographis japonica*, *Paris polyphylla* var. *yunnanensis*, and *Veratrum dahuricum* (Melanthiaceae).

A total of 510 601 protein-coding genes were annotated across these eight reference genomes based on functional databases (KEGG, GO, NR, KOG/COG, Swiss-Prot) [1, 3]. The platform harbors 0.82 Tb of transcriptomic data from 619 samples, encompassing 45 research projects and 13 distinct tissue types. This dataset is complemented by an interactive Transcriptome Atlas module for visualizing gene expression profiles. The platform also provides access to 102 chloroplast genomes (Fig. 1A).

**Figure 1:**
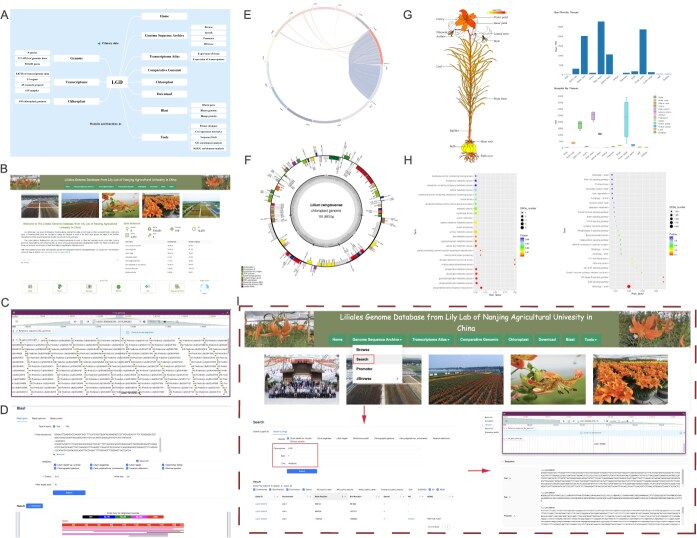
Overview of the main interface and internal features of LGD. (A) The datasets stored in LGD and the integrated tools in this database. (B) Homepage of LGD. (C) Overview of gene-level information (gene position, structure, and functional annotation). (D) Interface of the homology search implemented in LGD. (E) Syntenic blocks displayed in a circos plot. (F) Chloroplast genome display module. (G) Visual expression profile page. (H) Tools for GO and KEGG enrichment analysis. (I) Process of searching for genes and related information from specific chromosomal regions.

The LGD web interface and list of collected data support analyses centered around functional and comparative genomics, comprising eight main modules: Home, Genome Sequence Archives, Transcriptomic Atlas, Comparative Genomic, Chloroplast, Download, Blast, and Tools (Fig. 1B). These portals offer a wealth of user-friendly visualization resources, which were designed for the examination and comparison of genomic sequences, gene architectures, and expression profiles. Certain interfaces are dedicated to in-depth exploration of single genes, including information about their genome positions, expression level, and functional descriptions in the form of gene lists. Some tools enable a more comprehensive exploration of chromosome structure.

The Genome Sequence Archive module enables dynamic data visualization with four functional subcategories: Browser, Search, Promoter, and JBrowse (Fig. 1C and D). Each option provides access to distinct data retrieval tools. Browser displays a species-specific genomic overview; selecting a species reveals detailed gene annotations, including chromosomal locations, start/end positions, strand orientation, and functional descriptors (e.g. NR, GO, and KEGG terms). Search enables targeted queries using gene IDs or genomic coordinate ranges to extract precise gene records. Promoter provides access to the promoter sequences of all genes from Liliales species based on a simple gene ID search. JBrowse offers interactive visualizations of genomic features, including gene density across regions, exon–intron structures, and other locus-level details. All information associated with genes can be retrieved from the gene interface.

The Comparative Genomic module can be used to perform collinearity analysis on any two genomes to identify genomic syntenic blocks and syntenic gene pairs (Fig. 1E). A total of 10 582 genomic syntenic blocks were identified among or within the Liliales genome assembly sequences, containing 281 863 gene pairs. Among them, the proportion of syntenic block size to the whole genome size reached up to 97.88% for *L. davidii* var. *unicolor* and *L*. *sargentiae.*

The chloroplast module contains 102 complete chloroplast genome assemblies from the NCBI database, with sequence lengths ranging from 138 861 (*L. apertum*) to 153 164 (*L. souliei*) (Fig. 1F). All plastomes show a typical quadripartite structure, comprising a long single-copy region and a short single-copy region demarcated by two inverted repeat regions. The number of annotated genes is similar among the chloroplast genomes, ranging from 115 (*L. apertum*) to 132 (*L*. *lancifolium*).

The Transcriptomic Atlas module can be divided into two parts: tissue and transcriptome expression. Gene expression profiles can be visualized for 13 organs of *L. davidii* var. *unicolor* (Fig. 1G), along with retrieval of information from various transcriptome projects in NCBI and corresponding gene expression levels based on gene ID. We retrieved all publicly accessible RNA-Seq datasets of *Lilium* from the NCBI Sequence Read Archive (SRA) database, encompassing data from 45 research projects and 619 samples representing multiple stages of growth and development, associated bacteria, and various treatments (temperature, NaCl, or hormones). In addition, LGD summarizes the source data and related literature.

The Tools module provides various functions, including primer design, batch sequence extraction, co-expression network analysis, and gene enrichment analysis. Primer design can be carried out for specific chromosomal positions and target sequences. The co-expression analysis tool identifies functionally related gene modules from transcriptomic data across diverse tissues and conditions. Users can input a gene of interest to retrieve co-expressed genes and visualize their interaction networks, facilitating exploration of regulatory relationships and discovery of genes involved in specific biological processes. Gene enrichment analysis tools (GO and KEGG) are integrated in LGD to identify functional categories from a gene list of interest (Fig. 1H).

The Blast module provides integrated sequence alignment tools. Users can perform nucleotide–nucleotide or protein–protein alignment against a reference genome or gene set. Sequences can be input directly or uploaded via a file. Key parameters such as *e*-value and word size can be customized.

The Download module summarizes the assembly and annotation results for the reference genomes. Files containing genomes, coding sequences, and protein sequences are in FASTA format, whereas the annotation files are provided in GFF3 file format. Custom datasets can be exported using the ‘sequence fetch’ feature based on gene IDs or input sequence ranges. LGD also provides relevant references in the ‘cite’ section.

To enhance usability, a step-by-step workflow is provided for gene retrieval using the Gene Sequence Archive module, illustrated by three high-resolution screenshots (Fig. 1I). This flowchart demonstrates how to identify candidate genes within specific genomic regions, access detailed sequence information for specific gene IDs, and view tissue-specific expression profiles across various lily tissues, facilitating efficient screening of candidates expressed in particular tissues.

Unlike comprehensive databases with broad but nontargeted plant coverage, LGD focuses specifically on Liliales, integrating reference genome data of eight species to support related research. The transcriptome data obtained under various treatments and visualization of gene expression profiles for 13 organs of *L. davidii* var. *unicolor* enable gaining a comprehensive understanding of lily expression patterns. The additional chloroplast genome module with 102 assemblies from NCBI enriches data types, enhancing database integrity while boosting its practicality and application scope.

With the continuous emergence of new sequencing technologies and biotechnologies, along with the development of bioinformatics analysis techniques, an increasing number of Liliales plant genomes will be completed through whole-genome sequencing. LGD will continuously update and integrate available multi-omics data from Liliales plants, providing innovative and convenient analysis tools to lay the foundation for research into functional genomics, genetic breeding, and genomic evolution, which can in turn promote the development of molecular breeding for Liliales. We welcome contributions, insights, and suggestions from researchers for the continuous improvement of LGD.

## Data Availability

All the data hosted in the LGD are freely available at https://lgd.njau.edu.cn/lily/.

## References

[1] Xu S, Chen R, Zhang X. et al. The evolutionary tale of lilies: Giant genomes derived from transposon insertions and polyploidization. Innovation (Camb). 2024;5:100726 39529947 10.1016/j.xinn.2024.100726 PMC11551468

[2] Kuo YT, Câmara AS, Schubert V. et al. Holocentromeres can consist of merely a few megabase-sized satellite arrays. Nat Commun. 2023;14:3502 37311740 10.1038/s41467-023-38922-7 PMC10264360

[3] Sun J, Wang X, Wang K. et al. Genomic and epigenomic insight into giga-chromosome architecture and adaptive evolution of royal lily (*Lilium regale*). Nat Commun. 2025;16:5617 40595690 10.1038/s41467-025-61289-w PMC12214920

[4] Liang Y, Gao Q, Li F. et al. The giant genome of lily provides insights into the hybridization of cultivated lilies. Nat Commun. 2025;16:45 39747119 10.1038/s41467-024-55545-8 PMC11696169

[5] Landis JB, Harden JJ, Eifler E. et al. Reference genome of *calochortus tolmiei* hook. & arn. (Liliaceae), a cat’s ear mariposa lily. G3 (Bethesda). 2025;15:jkaf008 39829026 10.1093/g3journal/jkaf008 PMC11917488

[6] Zeng P, Zong H, Han Y. et al. Two melanthiaceae genomes with dramatic size difference provide insights into giant genome evolution and maintenance. Nat Plants. 2025;11:1500–13 40750694 10.1038/s41477-025-02060-3

[7] Wu T, Liu Z, Yu T. et al. Flowering genes identification, network analysis, and database construction for 837 plants. Hortic Res. 2024;11:uhae013 38585015 10.1093/hr/uhae013 PMC10995624

[8] Ye J, Wang C, Liu Y. et al. CGD: a multi-omics database for chrysanthemum genomic and biological research. Hortic Res. 2024;11:uhae238 39512782 10.1093/hr/uhae238 PMC11541226

[9] Liu Z, Zhang C, He J. et al. plantGIR: a genomic database of plants. Hortic Res. 2024;11:uhae342 39712867 10.1093/hr/uhae342 PMC11661351

